# Activation of lactate receptor HCAR1 down-modulates neuronal activity in rodent and human brain tissue

**DOI:** 10.1177/0271678X221080324

**Published:** 2022-03-03

**Authors:** Marc Briquet, Anne-Bérengère Rocher, Maxime Alessandri, Nadia Rosenberg, Haissa de Castro Abrantes, Joel Wellbourne-Wood, Céline Schmuziger, Vanessa Ginet, Julien Puyal, Etienne Pralong, Roy Thomas Daniel, Stefan Offermanns, Jean-Yves Chatton

**Affiliations:** 1 Department of Fundamental Neurosciences, University of Lausanne, Lausanne, Switzerland; 2 Department of Neurosurgery Service, University Hospital of Lausanne and Faculty of Biology and Medicine, UNIL, Lausanne, Switzerland; 3 Max Planck Institute for Heart and Lung Research, Bad Nauheim, Germany; 4 Cellular Imaging Facility, University of Lausanne, Lausanne, Switzerland

**Keywords:** Dentate gyrus, electrophysiology, epilepsy, HCA1 receptor, human brain slices

## Abstract

Lactate can be used by neurons as an energy substrate to support their activity. Evidence suggests that lactate also acts on a metabotropic receptor called HCAR1, first described in the adipose tissue. Whether HCAR1 also modulates neuronal circuits remains unclear. In this study, using qRT-PCR, we show that HCAR1 is present in the human brain of epileptic patients who underwent resective surgery. In brain slices from these patients, pharmacological HCAR1 activation using a non-metabolized agonist decreased the frequency of both spontaneous neuronal Ca^2+^ spiking and excitatory post-synaptic currents (sEPSCs). In mouse brains, we found HCAR1 expression in different regions using a fluorescent reporter mouse line and *in situ* hybridization. In the dentate gyrus, HCAR1 is mainly present in mossy cells, key players in the hippocampal excitatory circuitry and known to be involved in temporal lobe epilepsy. By using whole-cell patch clamp recordings in mouse and rat slices, we found that HCAR1 activation causes a decrease in excitability, sEPSCs, and miniature EPSCs frequency of granule cells, the main output of mossy cells. Overall, we propose that lactate can be considered a neuromodulator decreasing synaptic activity in human and rodent brains, which makes HCAR1 an attractive target for the treatment of epilepsy.

## Introduction

Lactate is recognized as an energy substrate for neurons.^1,2^ It can be provided to neurons by transport from astrocytes to the extracellular space, thereafter entering neurons through monocarboxylate transporters (MCTs),^3^ as well as from the blood stream. The extracellular brain lactate level is estimated to be in the low millimolar range at resting state^4,5^ and to undergo a two-fold increase during synaptic activity.^6^ During intense physical exercise, plasma lactate, which can cross the blood-brain barrier, can rise to 10–20 mM.^7^ Besides this metabolic role, lactate, while in the extracellular space, may exert other actions on brain cells,^8^ notably for long-term memory formation.^9–11^ Studies have reported that lactate can also influence the excitability of select populations of neurons via different metabolic pathways. Lactate was found to change the firing frequency of glucose-sensing neurons of hypothalamic and GABAergic neurons of the subfornical organ,^12^ the center for the control of salt intake behavior. Studies have also demonstrated that lactate influences the activity of glucose-sensitive ventromedial hypothalamic neurons^13^ and orexin neurons.^14^ Lactate was shown to be necessary for inducing plasticity-related genes expression in neurons through potentiation of NMDA receptor activity.^15^ Evidence for L-lactate-mediated excitation of neuronal activity triggering norepinephrine release was described in the locus coeruleus.^16^ These findings raise the question of whether receptor-mediated non-metabolic mechanisms of lactate could underlie these effects in the brain. This would confer additional roles on lactate such as that of a signaling molecule for neurons.^3,17^

In this context, a new family of G-protein coupled receptors (GPCR) called hydroxycarboxylic acid receptors (HCAR) has recently been identified in adipocytes.^18^ HCARs are activated by several intermediates of cellular energy metabolism. Among them, HCAR1 (named GPR81 before being deorphanized) is considered to be a sensor for lactate in peripheral organs.^19,20^ HCARs are reported to be coupled to G_i_ proteins^20^ and their activation has antilipolytic effects.^21–23^ Recently, we and other labs have demonstrated that HCAR1 is present in brain cells.^23–25^ HCAR1 expression was found to be enhanced in models of ischemic stroke 24 hours after reperfusion,^26^ which was accompanied with a reduction of cell death.

However, because current commercial HCAR1 antibodies lack specificity,^27,28^ the precise expression and localization of HCAR1 in the brain is still ambiguous. At the functional level, our group confirmed that HCAR1 is active at the membrane of cultured primary cortical neurons.^24,27^ Its activation by lactate was concentration-dependent and was not consistent with a metabolic effect, considering that it was not observed with glucose and even the closely related metabolite pyruvate. Lactate and non-metabolized agonists of HCAR1 caused a reversible, concentration-dependent decrease in spiking activity, an effect that was absent in HCAR1-deficient mice (HCAR1 KO). The intracellular mechanisms involves the inhibition of the adenylyl cyclase – cAMP – protein kinase A axis which leads to a decreased synaptic vesicular release and reduction of excitability,^27^ which was also reported in rat hippocampal CA1 neurons.^29^

In this study, we investigated the specific localization and function of HCAR1 in brain tissue. We report that HCAR1 is expressed in the human and mouse brain. We used HCAR1 fluorescent reporter mice and new *in situ* hybridization technology to map the expression of HCAR1 in the mouse brain in specific neuronal population including sub-regions of the hippocampus and cerebellum. At the functional level, we show a neuromodulation caused by HCAR1 activation in the dentate gyrus (DG) of the hippocampus in mouse and rat. Importantly, this modulation is also present in fresh human cortical slices. Indeed, HCAR1 activation by non-metabolic ligands cause a decrease in firing and spontaneous excitatory postsynaptic current (sEPSCs) frequency. We therefore demonstrate that HCAR1 drives negative metabolic feedback on neuronal activity *in situ* both in rodent brain tissue and in human epileptic tissue.

### Material and methods

#### Human tissue experiments

Human tissue was made available for experiments within an experimental framework approved by the Cantonal Ethics Committee on human research (CER-VD, protocol number 207/10) and procedures. Brain tissue samples resected from patients undergoing surgery at the Service of Neurosurgery of Lausanne University Hospital (CHUV) were used for in vitro neuronal activity recordings. Patients provided written informed consent to participate in the study, which operated in accordance with the ethical standards of the institutional national research committee and with the 1964 Helsinki declaration and its later amendments.

Human tissue is classified as ‘epileptic' as it originated from resected tissue of the epileptogenic locus of patients with dysplasia (or other forms of epilepsy) and who were pharmacoresistant. Brain samples were from both male (57%) and female (43%) patients with median age 18 years, and originated from frontal (n = 5), insular (n = 2), parietal (n = 1), and temporal (n = 9) cortex from both hemispheres.

#### Animal tissue experiments

All animal experimentation procedures were carried out in accordance with the recommendations of the Swiss Ordinance on Animal Experimentation, and were specifically approved for this study by the Veterinary Affair of the Canton Vaud, Switzerland (authorizations# VD1288.6-7-8 and VD2927d) and conformed to the ARRIVE guidelines. Animals were housed at our local animal facility with ad libitum access to food and water (maximum 5 animals per cage) before euthanasia for brain tissue preparation. Male C57BL/6N and HCAR1 KO^21^ mice (18–25 days old) were used for experiments. Transgenic mice (male 20 days old) that expresses monomeric red fluorescent protein (mRFP) under the HCAR1 promoter generated and validated as previously described was used for HCAR1 localization.^21^ This fluorescent reporter protein is not targeted to the plasma membrane but spreads in the cytoplasm, allowing us to identify the cells which endogenously express the HCAR1 transcripts. Male Sprague Dawley (30–35 days old) were used for experiments.

#### qRT-PCR

Human tissue from patient and dissected brain from C57BL/6N mice at P7 (n = 3), P14 (n = 3), and P31 (n = 6) were instantaneously frozen in liquid nitrogen. In another group of P31 mice, brain was further dissected out into different brain regions (cerebellum, hippocampus, brainstem and cortex). Extraction and purification of RNA were done using the RNeasy Mini Kit (Qiagen, Basel, Switzerland, n°74104) following the protocol from the manufacturer. RNA assay and analysis was performed with Agilent RNA 6000Nano Kit (Agilent Technologies, Santa Clara, Ca, USA, n°5067-1511). Reverse transcriptase was carried out with High-Capacity cDNA Reverse Transcription Kit (Applied biosystems, Ca, USA, n°4368814). qPCR for hydroxy-carboxylic acid receptor 1 (HCAR1) was normalized with the housekeeping gene β-actin for the human tissue and GAPDH for the mouse tissue. Primer pair sequences (Supplementary Table 1) was carried out in the CFX Connect™ Real-Time System (BioRad, California, USA) using the iQ SYBR® Green Supermix 2x (Bio-Rad, n°1708880) for the human tissue and in the CFX96 Touch Real-time PCR detection system (BioRad) using the Power SYBR Green PCR Master mix (BioRad) for the mouse tissue. All samples were run in triplicate. The homogeneity of the triplicates was assessed calculating the percentage of difference between them [(SD/mean)*100] and the ones that had a difference bigger than 2% were eliminated. For analysis, all Cq values were rescaled for each gene to the lowest Cq value as an internal control, converted these rescaled Cq logarithmically into linear, relative quantities taking into account the gene specific amplification efficiency [relative quantity = 1 + efficiency^(Cqinternal control − Cqsample)^]. Finally, arithmetical means were obtained as means from the triplicates. Expression of HCAR1 was then normalized to the reference gene.

#### Immunohistochemistry

mRFP mice were anesthetized with sodium-pentobarbital (150 mg/kg, intraperitoneally) and transcardially perfused with fresh solutions of 4% paraformaldehyde (PFA) in phosphate buffered saline (PBS) 0.1 M (pH 7.4) for 10 min. Brain extraction was followed with 24 h post-fixation at 4°C with 4% PFA. Afterwards, both hemispheres were sliced in PBS at 50 μm with a vibratome (Leica, VT1000S).

Fixed slices were then rinsed 3 times in PBS prior to a 2-hour incubation in blocking solution (10% donkey serum and 0.1% Triton X-100 diluted in PBS) at room temperature (RT). Slices were then incubated overnight (O/N) at 4°C with primary antibodies (Table 1). Next, slices were washed 3 times in PBS and incubated 2 hours at RT with respective secondary antibodies. For nuclei staining, Hoechst (2 µg/µl) a 10-minute incubation was performed. Finally, stained slices were mounted on slides using FluorSave mounting medium (Merck-Millipore, Darmstadt, Germany).

**Table 1.** Primary and secondary antibodies used in the study.

	Source	Catalogue number	Concentration
Primary antibodies
Rabbit anti-mRFP	Rockland Immunochemicals	600-401-379	1:1000
Rat anti-mRFP	Chromotek	AB_2336064	1:100
Rabbit anti-GluR2/3	Merck-Millipore	07-598	1:400
Mouse anti-GFAP	Sigma-Aldrich	G3893	1:200
Mouse anti-NeuN	Merck-Millipore	MAB377	1:200
Mouse anti-GAD67	MAB5406 Merck-Millipore	MAB5406	1:1000
Secondary antibodies			
Donkey anti-rabbit IgG Alexa Fluor 594	Invitrogen	A21207	1:200
Donkey anti-mouse IgG Alexa Fluor 488	Invitrogen	R37114	1:200
Donkey anti-rat IgG Alexa Fluor 488	Invitrogen	A48269	1:200

Imaging of mRFP stained sections was performed using a Zeiss LSM780 confocal laser scanning microscope (Zeiss, Oberkochen, Germany) outfitted with a 63X 1.40 NA Plan Apochromat oil immersion objective lens.

#### Two-photon imaging

Two-photon imaging of endogenously expressed mRFP in mouse acute brain slices of 300 µm was carried out using a custom-built two-photon microscope with a 40X 0.8 N.A. water-dipping objective (Olympus, Tokyo, Japan). Fluorescence excitation was performed using a Chameleon Vision S femtosecond infrared laser including group velocity dispersion compensation (Coherent, Ca, USA). Captured emission wavelengths were 607 ± 70nm for red channel. Image acquisition was performed using custom-written software in the Labview environment. ImageJ software (RRID:SCR_003070) was further used for downstream image processing.

#### In situ hybridization – RNAscope™

C57BL/6N mice were deeply anesthetized with sodium-pentobarbital and transcardially perfused with 50 ml of 4% PFA in 0.1 M PBS (pH 7.4). Brains were dissected out and post-fixed in 4% PFA for 24 h at 4°C. The tissues were then washed 3 times with PBS and cryoprotected in 10% (O/N at 4°C), 20% (O/N at 4°C) and 30% (O/N at 4°C) sucrose in PBS. Tissues were embedded in Tissue-TEK O.C.T. compound (Sakura Finetek, Torrence, Ca, USA) sectioned at 14 µm with a cryostat (Leica, CM3050 S), and mounted onto Superfrost Ultra Plus slides (Thermo Fischer Scientific, Waltham, MA, USA). For *in situ* hybridization (RNAscope^TM^, Advanced Cell Diagnostics, San Francisco, CA, USA), the manufacturer’s protocol was followed. All experiments were replicated in three animals. The probes were designed by the manufacturer and available from Advanced Cell Diagnostics. The following probe was used in this study: Mm-GPR81-C1 (#4317421).

#### Human: Electrophysiology and calcium imaging

After resection, cortical tissue was quickly placed in ice-cold artificial CSF (aCSF) slicing solution continuously bubbled with 95% O_2_/5% CO_2_ and that contained (mM): 110 choline chloride, 26 NaHCO_3_, 10 D-glucose, 11.6 ascorbic-acid, 7 MgCl_2_, 3.1 Na-pyruvate, 2.5 KCl, 1.25 NaH_2_PO_4_, and 0.5 CaCl_2._^30^ The tissue was then swiftly transported to the neurophysiology laboratory. Transition time from tissue resection to slice preparation was approximately 10 min. Tissue was placed in a petri dish containing ice-cold and oxygenated slicing solution for removal of the pia, if necessary, and orientation in order to slice perpendicular to the white matter. The tissue blocks were then glued on mounting plate and submerged in slicing chamber filled with ice-cold slicing solution. Slices are cut at 340 μm and then transferred to a holding chamber in which they were stabilized for 20 min at 34°C. Subsequently, slices were stored for at least 1 h at RT before recording in aCSF containing the following (mM): 125 NaCl, 26 NaHCO_3_, 3 KCl, 1.25 NaH_2_PO_4_, 1 MgSO_4_, 2 CaCl_2_, 3 myo-inositol, 3 Na-pyruvate, 0.5 ascorbic acid, and 10 glucose. Recording aCSF solution was similar to holding solution with glucose reduced to 2.5 mM.

For recording, each slice was transferred in a double perfusion recording chamber submerged in recording aCSF (34°C) and attached to the stage of a Zeiss LSM510 Meta upright microscope equipped with infrared differential interference contrast and 40X water dipping objective. Whole-cell patch-clamp recordings were made from pyramidal cells using standard ∼5 MΩ borosilicate glass pipettes filled with the internal solution containing (mM): 130 K-gluconate, 5 KCl, 5 NaCl, 1 MgCl_2_, 0.1 EGTA, 0.025 CaCl_2_, 10 HEPES; 5 Na-phosphocreatine; 4 Glucose, 4 ATP-Mg; 0.3 GTP-Na, biocytin 1 mg/ml (pH = 7.3 adjusted with KOH, 290mOsm). Recordings were obtained in voltage- and current-clamp configuration with a Multiclamp 700B amplifier (Molecular Devices, San José, CA, USA). Data were acquired with a Digidata 1440 A (Molecular Devices), at 10 kHz sampling rate and filtered at 2 kHz, controlled with pCLAMP 10 software (RRID:SCR_011323). Access resistance was monitored by a −5mV step (0.1 Hz). Experiments were discarded if the access resistance varied by more than 20%. Spontaneous excitatory post-synaptic events (sEPSCs) were recorded for 2 min from a holding potential of −80mV in the absence and in the presence of 3Cl-HBA (40 µM). Stable (otherwise rejected) sEPSCs recordings were manually analyzed offline using the MiniAnalysis software (Synaptosoft Inc, USA, RRID:SCR_002184). For each cell, the frequency and the amplitude of these synaptic events were analyzed. We further assessed cell passive properties and firing frequency in control conditions and after HCAR1 activation. A series of positive and negative current steps (30 pA increments, starting from −120pA) of 2000 ms duration were injected to measure the firing frequency. Action potential (AP) frequency was measured as the number of AP in response to 540 pA current injection. Input resistance was determined by passing current steps (2000 ms, with 30 pA increments from −120 to 0 pA), and calculated as the slope of the current-voltage plot. The rheobase was determined as the minimal current amplitude able to evoke an AP, and was obtained by applying 3-sec steps of positive current (starting at 0 pA, 50 pA increments). These cell passive properties and firing frequency were recorded control conditions (baseline) and after HCAR1 activation. At the end of recordings, slices were fixed and processed with streptavidin-AlexaFluor 647 (1:500, Invitrogen, Catalog # S21374) for morphological characterization.

Neuronal activity was also monitored by calcium imaging using the membrane-permeant dyes Fluo-4 AM (Teflabs, Austin, TX, USA) or Fluo-8 AM (Abcam, Cambridge, UK), diluted at 1 mM in DMSO and applied by bolus loading using a pressure ejection system (Picospritzer II, General Valve, or Toohey Company) imaged by wide-field fluorescence or two-photon microscopy. Widefield calcium imaging was performed with an upright epifluorescence microscope (FN1, Nikon, Tokyo, 163 Japan) using a 40X 0.8 N.A water-immersion objective lens. Fluorescence excitation wavelengths were selected using a fast filter wheel (Sutter Instr., Novato, CA) and fluorescence was detected using an Evolve EMCDD camera (Photometrics, Tucson, AZ, USA). Digital image acquisition and time series were computer-controlled using the Metafluor software (RRID:SCR_014294). For two-photon imaging, we used a custom-built multiphoton microscope equipped with a 40X 0.8 N.A. objective (Olympus) and a femtosecond Ti:Sapphire laser (Chameleon Vision S, Coherent) with excitation at 820 nm. Fluorescence intensity over time was recorded at 1–2 Hz and ultimately assessed using a custom image analysis software by placing regions-of-interest (ROIs) over neuronal somata.

#### Mouse: Electrophysiology

In anesthetized C57BL/6N and HCAR1 KO mice, brains were removed promptly after decapitation and submerged in ice-cold aCSF slicing solution containing (in mM): 86 NaCl, 75 sucrose, 25 NaHCO_3_, 25 D-glucose, 4 MgCl_2_, 3 KCl, 1 NaH_2_PO_4_, 1 CaCl_2_. All aCSF solutions were continuously bubbled with 95% O_2_/5%CO_2_. 300 μm thick sagittal acute slices were prepared using a vibratome (Leica VT-1000S). Slices were then transferred to holding chambers in oxygenated slicing solution for 15 min at 34°C. Slices were then placed for 1 h at RT in an oxygenated incubation solution containing (mM): 120 NaCl, 26 NaHCO_3_, 5 D-glucose, 1 MgSO_4_, 3.2 KCl, 1 NaH_2_PO_4_, 2 CaCl_2_.

For recording, each slice was transferred in the same setup described above and continuously superfused with oxygenated recording solution containing (mM): NaCl, 26 NaHCO_3_, 10 Na-oxamate, 5 Na-pyruvate, 2.5 D-glucose, 1 MgSO_4_, 3.2 KCl, 1 NaH_2_PO_4_, 2 CaCl_2_. Cells visualized in the granule cell layer of the dentate gyrus were targeted for recording in whole-cell configuration with borosilicate glass pipettes (6–8 MΩ) filled with internal solution containing (mM): 130 K-gluconate, 5 KCl, 5 NaCl, 1 MgCl_2_, 0.1 EGTA, 0.025 CaCl_2_, 10 HEPES; 5 Na-phosphocreatine; 4 glucose, 4 ATP-Mg; 0.3 GTP, biocytin 1 mg/ml (pH = 7.3 adjusted with KOH, 290mOsm). Experiments were discarded if the access resistance varied by more than 20%. sEPSCs were recorded for 3 min from a holding potential of −70mV in the absence and in the presence of 3Cl-HBA (40 µM). mEPSCs were pharmacologically isolated by having picrotoxin (100 μM) (Tocris, Bristol, UK, Catalog # 1128) and tetrodotoxin (TTX, 1 μM) (Alomone labs, Jerusalem, Israel, Catalog # T-550) present throughout the experiment while clamping the cells at −70 mV. Analyses of sEPSC and mEPSC were performed offline and verified by eye using the MiniAnalysis software. For each cell, the frequency and the amplitude of these synaptic events were analyzed. To investigate the excitability, the maximum firing frequency reached in each condition was quantified, as well as the necessary current injected to produce this maximum firing. *R*_N_, RMP, and rheobase were acquired and analyzed as described above. At the end of recordings, slices were fixed and processed with streptavidin-AlexaFluor 647 for cell identification and morphological characterization.

#### Rat: Electrophysiology

Sprague Dawley rats were anesthetized with isoflurane and rapidly decapitated. Once removed, brain was placed in ice-cold aCSF slicing solution containing (in mM): 210 sucrose, 2.8 KCl, 2 MgSO_4_, 1.25 Na_2_HPO_4_, 25 NaHCO_3_, 1 MgCl_2_, 1 CaCl_2_ and 10 D-Glucose.^29^ All aCSF solutions were continuously bubbled with 95% O_2_/5%CO_2_. 385 μm thick horizontal acute slices of each hemisphere were prepared using a vibratome (Leica VT-1000S). Slices were then transferred to a holding chamber for 30 min at 34°C in oxygenated aCSF containing (in mM): 125 NaCl, 2.5 KCl, 1.25 Na_2_HPO_4_, 25 NaHCO_3_, 4 MgCl_2_, 1 CaCl_2_ and 10 glucose.^29^ Slices were then placed for 90 min at RT before any experimental procedure.

For recording, each slice was transferred in the same setup as described for mouse experiments and continuously superfused with oxygenated recording solution containing (mM): 125 NaCl, 2.5 KCl, 1.25 Na_2_HPO_4_, 25 NaHCO_3_, 2 MgCl_2_, 2 CaCl_2_ and 10 D-glucose, pH = 7.35–7.4. Cells visualized in the granule cell layer of the dentate gyrus were targeted for recording in whole-cell configuration with borosilicate glass pipettes (6-8MΩ) filled with internal solution containing (mM): 135 K-gluconate, 10 KCl, 5 NaCl, 1 EGTA, 10 HEPES, 10 phosphocreatine; 2 Mg-ATP, 0.4 Na-GTP, biocytin 1 mg/ml (pH = 7.3 adjusted with KCl, 290mOsm). Experiments were discarded if the access resistance varied by more than 20%. sEPSCs were recorded for 3 min from a holding potential of −70mV in the absence and in the presence of 3Cl-HBA (40 µM). Analyses of sEPSC were performed offline and verified by eye using the MiniAnalysis software. For each cell, the frequency and the amplitude of these synaptic events were analyzed. Excitability and intrinsic properties were analyzed the same way as the mouse one. At the end of recording, slices were fixed and processed with streptavidin-Alexa Fluor 647 for cell identification and morphological characterization.

#### Experimental design, data analysis, and statistical significance

Data analysis was performed using Clampfit (Molecular Devices, RRID:SCR_011323) and Prism (GraphPad, San Diego, USA; RRID:SCR_002798). Most data are represented using scatter plots showing mean ± SD and individuals values. We employed the following statistical tests: Student’s t test, one sample t test (when compared to the percentage of baseline) and one-way repeated measure ANOVA. Unless otherwise specified, tests are paired and two-tailed. Parametric or non-parametric tests were performed according to data normality verified with the Shapiro-Wilk test. *Post hoc* corrections for multiple comparisons were performed when appropriate (after one-way ANOVA). Significance was conventionally set as ∗∗∗*P* < 0.001, ∗∗*P* < 0.01 and *P < 0.05.

#### Chemicals and drugs

3Cl-HBA was obtained from Sigma-Aldrich (Catalog #16795) or Cayman chemicals (Catalog #16795). Other chemicals were from Sigma-Aldrich.

## Results

### Characterization of HCAR1 brain expression

Limited data is available on HCAR1 expression and functions in the brain. In addition, the reported non-specificity of HCAR1 antibodies led us to select different approaches to identify HCAR1-expressing cells in the mouse brain.^27^

**Figure 1.**
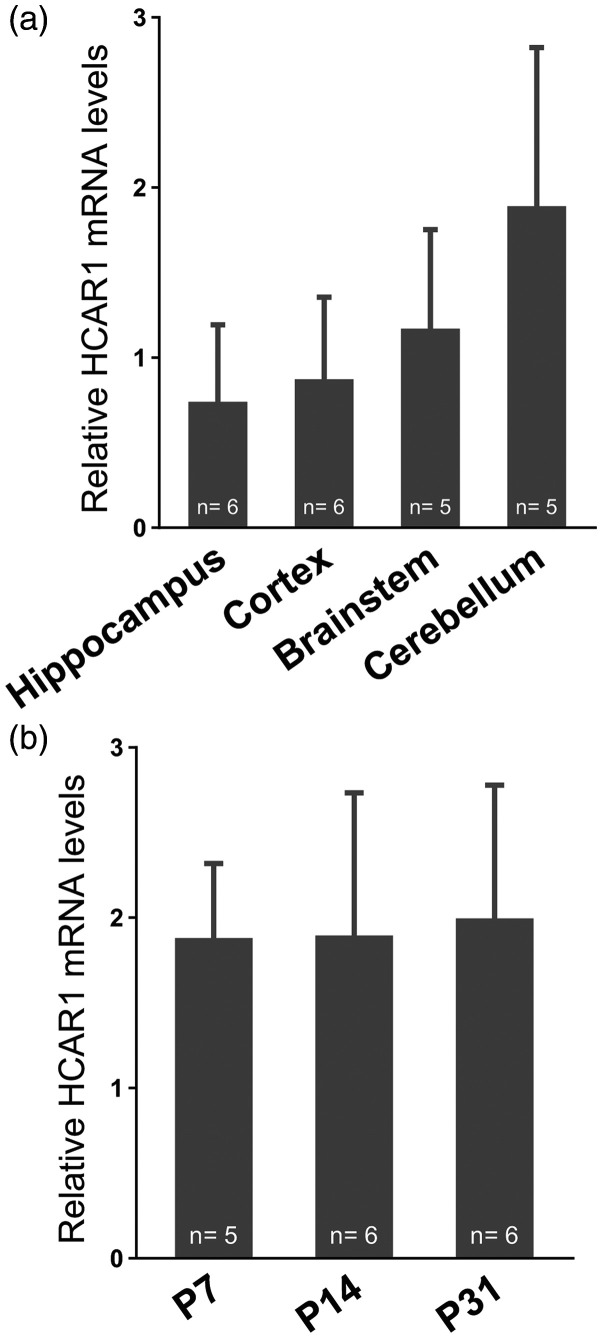
HCAR1 mRNA transcript expression in different mouse brain regions and across age. (a) HCAR1 mRNA detection in selected regions after dissection of 1-month old mice. (b) HCAR1 mRNA detection in brains from mice at post-natal day 7, 14, 31. Results are expressed relative to GAPDH expression as means ± SD. The number of experiments are indicated in the graph.

First, we looked for the presence of HCAR1 mRNA using qRT-PCR. Figure 1(a) shows that HCAR1 mRNA was found in hippocampus, cortex, brain stem, and cerebellum of wild-type mice. Hemi-brain samples prepared from mice at different ages (P7, P14, and P31) displayed the same levels of HCAR1 transcripts, which indicates that HCAR1 transcription is not age-dependent (Figure 1(b), *P =* 0.95, one-way ANOVA). As expected, no cDNA could be amplified from brain samples of HCAR1 KO mice (Supplementary Figure 1).

**Figure 2.**
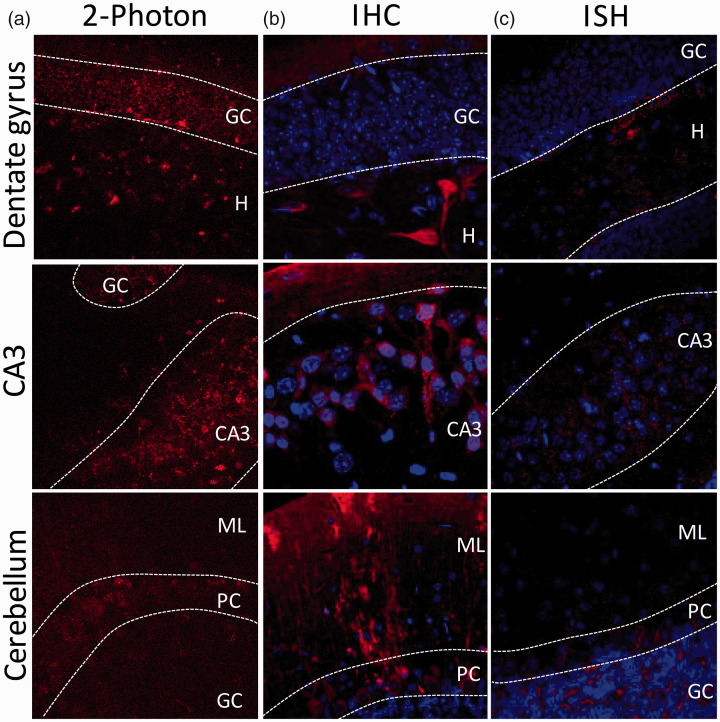
HCAR1 positive cells in dentate gyrus, CA3 and cerebellum. Analysis of brain tissue from reporter mouse line expressing mRFP under the HCAR1 promoter revealed scattered mRFP-positive cells in several regions of the brain. (a) Using a 2-photon microscope, endogenous mRFP signal on fresh 300 µm acute slice was found in the GC layer and the hilus of the DG, in the CA3, and in the Purkinje cell layer of the cerebellum. (b) Using anti-mRFP immunohistochemistry to reveal HCAR1-mRFP positive cells, mRFP expression was found in the hilus of the DG as well as in the inner molecular layer, in the CA3, and in the Purkinje, molecular, and granular cell layer of the cerebellum. Alexa 680 secondary antibody staining is shown in red and nuclei (Hoechst) in blue. (c) Using in situ hybridization (RNAscope™), HCAR1 mRNA transcript was found in the hilus and in the GC layer of the DG, in the CA3, and mainly in the granule and Purkinje cell layer. HCAR1 transcript is shown in red and nuclei (DAPI) in blue. GC = granule cell layer, H = hilus, ML = molecular layer, PC = Purkinje cell.

**Figure 3.**
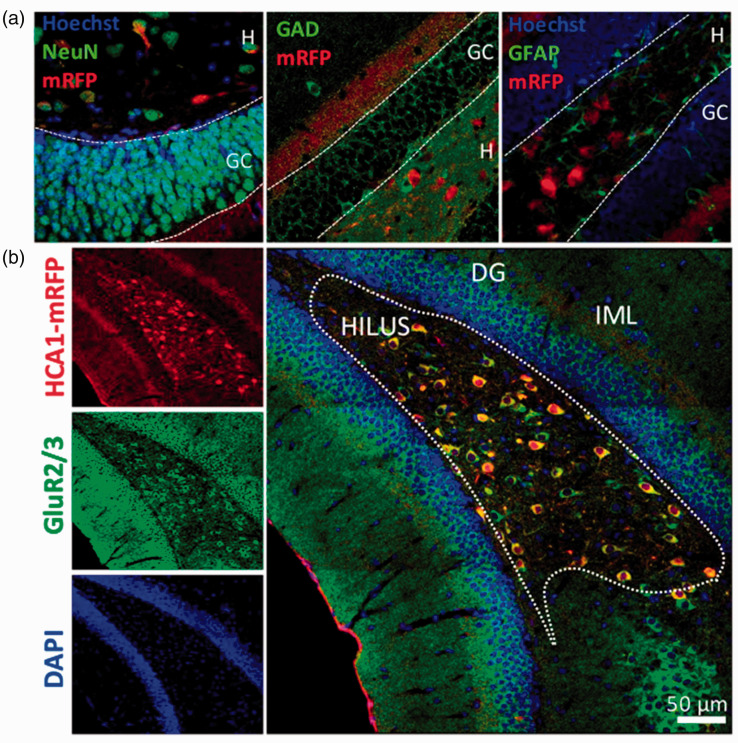
Identity and distribution of HCAR1 positive cells in the dentate gyrus. Hippocampal mRFP-positive cells were found mainly in the DG. (a) These cells colocalized with the neuronal marker NeuN, but not with the GABAergic cell marker GAD67, nor with the glial GFAP marker. (b) In the hilar region of the DG, mRFP-positive cells colocalized with the maker GluR2/3 shown to be associated with MCs. GC = granule cell layer, H = hilus, IML = inner molecular layer.

The distribution of HCAR1 expressing cells was then investigated using a reporter mouse line expressing mRFP under the HCAR1 promoter.^21^ Results suggest that HCAR1 is expressed in specific brain regions of the mRFP reporter mice. Using 2-photon imaging of endogenous mRFP signal in fresh mouse acute brain slices (Figure 2(a)), we found expression of mRFP in the granule cell (GC) layer and the hilus of the DG, in the CA3 region, and in the cerebellum. We then acquired immunofluorescent confocal images of mRFP signal to enable counterstaining of cell nuclei. HCAR1 positive cells are found in the hippocampus and the cerebellum. Hippocampal expression is highest in the hilar area of the DG and in the pyramidal cells from CA3 (Figure 2(b)). In the cerebellum, the Purkinje cell (PC) layer and the molecular layer have the strongest expression. Finally, we carried out *in situ* hybridization assays (Figure 2(c)). In the hippocampus, HCAR1 transcripts were observed throughout the DG, while more abundantly expressed in the hilus and in the CA3 region. In the cerebellum, HCAR1 transcript was found in the GC and the PC layers. To further phenotype the cellular expression of HCAR1 in the DG, our main region of interest, co-staining with cellular markers was performed (Figure 3(a)). We confirmed the neuronal identity of HCAR1-mRFP positive cell using the neuronal marker NeuN. The absence of colocalization with GAD67 and GFAP staining indicates that mRFP positive cell are not hilar GABAergic neurons and astroglial cells. HCAR1-positive cells of the hilus colocalized with GluR2/3, a specific marker for the mossy cells (MCs) in the DG^31^ (Figure 3(b)). This specific hilar HCAR1 expression pattern prompted to further explore its functional role in the DG network.

### HCAR1 influences synaptic events at GCs synapses in the mouse brain

First, we used organotypic hippocampal slices to assess HCAR1-dependent neuronal activity modulation. By using calcium imaging, we observed a down-modulation of neuronal activity by 42% (Supplementary Figure 2; n = 107 cells, *P <* 0.0001, one-way ANOVA) after HCAR1 activation by 3Cl-HBA, a non-metabolized HCAR1 agonist.^22^

**Figure 4.**
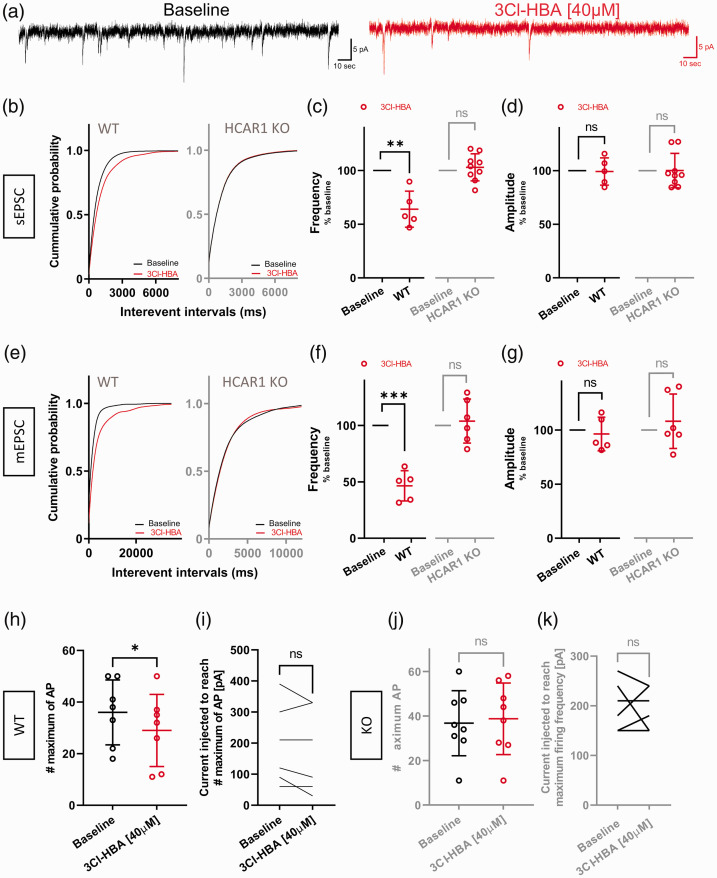
Granule cells from mice decrease spontaneous and mini EPSCs and firing frequency after activation of HCAR1. (a) Example traces of sEPSCs measured during baseline condition (black) and following application of 40 µM 3Cl-HBA (red). (b) Cumulative distributions of sEPSCs frequency in WT and HCAR1 KO were analyzed by using Kolmogorov-Smirnov test. WT: *P* < 0.0001 versus HCAR1 activation; HCAR1 KO: *P* = *P* = 0.18 versus HCAR1 activation (c) Summary of recorded cells showing a decreased frequency of sEPSC by 36% induced by 3Cl-HBA compared to baseline (one sample t, n = 5, mean = 63.97 ± 16.79, *P* = 0.0087). HCAR1 KO recorded cells showed no significant decrease in sEPSC frequency after 3Cl-HBA (one sample t, n = 9, mean = 103 ± 12.58, *P* = 0.5) (d) sEPSC amplitude showed no statistically significant changes between baseline and 3Cl-HBA application in both WT and HCAR1 KO (WT: one sample t, n = 5, mean = 99.22 ± 12.72, *P* = 0.9; HCAR1 KO: one sample t, n = 9, mean = 100.1 ± 16.07, *P* = 0.98). (e) Cumulative distributions of mEPSCs frequency in WT and HCAR1 KO were analyzed by using Kolmogorov-Smirnov test. WT: *P* < 0,0001 versus HCAR1 activation; HCAR1 KO: *P =* 0.14 versus HCAR1 activation. (f) Summary of recorded cells showing a decreased frequency of mEPSC by 53% induced by 3Cl-HBA compared to baseline (one sample t, n = 5, mean = 46.5 ± 13.42, *P* = 0.0009). HCAR1 KO recorded cells showed no significant decrease in mEPSC frequency after 3Cl-HBA (one sample t, n = 6, mean = 103.9 ± 19.55, *P* = 0.64) (g) mEPSC amplitude showed no statistically significant changes between baseline and 3Cl-HBA application both in WT and HCAR1 KO (WT: one sample t, n = 5, mean = 96.34 ± 15.52, *P* = 0.63; HCAR1 KO: one sample t test, n = 6, mean = 108.1 ± 25.10, *P* = 0.47). (h, i) Effect of HCAR1 activation on neuronal firing frequency following steps of current injection in WT mice. The maximum number of action potentials evoked was significantly decreased by HCAR1 activation (paired t test, n = 7, Baseline: mean = 36 ± 12.58, 3Cl-HBA: mean = 29 ± 13.98, *P* = 0.019), but not the current injected to reach the maximum firing frequency (paired t test, n = 7, Baseline: mean = 184.3 ± 121.8, 3Cl-HBA: mean = 162.9 ± 127.1, *P* = 0.14). (j, k) In HCAR1 KO, activation of HCAR1 did not change the maximum number of AP compared to baseline (paired t test, n = 8, Baseline: mean = 36.75 ± 14.62, 3Cl-HBA: mean = 38.75 ± 16.06, *P* = 0.62), nor the current injected to reach the maximum firing frequency (paired t test, n = 8, Baseline: mean = 202.5 ± 41.66, 3Cl-HBA: mean = 198.8 ± 35.63, *P* = 0.82). Values are means±SD, *P < 0.05, **P < 0.01, ***P < 0.001 versus 3Cl-HBA application.

In the DG, MCs mediate an intrinsic excitatory loop, receiving powerful inputs from a relatively small number of GCs and providing highly distributed excitatory outputs to a large numbers of GCs.^32^ As we found localization of HCAR1 mainly in MCs (Figure 3(b)), we hypothesized that HCAR1 activation by 3Cl-HBA may modulate synaptic events at GCs. We thus decided to dissect the functional role of HCAR1 in the DG network of acute slices by performing whole-cell patch clamp recordings of GCs, which receive inputs from MCs. We first recorded sEPSC from neurons of the GC layer (Figure 4(a)). We found that HCAR1 activation significantly altered the frequency of events, as indicated by a rightward shift in interevent cumulative probability distributions (Figure 4(b); *P* < 0.0001, K-S test). The mean frequency was decreased by 36% (Figure 4(c); *P* = 0.0087, one sample t test), but not the amplitude (Figure 4(d); *P* = 0.9, one sample t test). Importantly, this modulation was not observed in cells from HCAR1 KO mice (Figure 4(b), *P* = 0.18, K-S test; Figure 4(c), *P* = 0,5, one sample t test; Figure 4(d); *P* = 0.98, one sample t test), highlighting the specificity of 3Cl-HBA effects.

In addition, MCs also make synapses with GABAergic interneurons, which mediate feed-forward inhibition onto GCs.^32^ Therefore, to focus on the effect of HCAR1 activation on MC-GC synapses, we recorded miniature excitatory post-synaptic currents (mEPSC) in the presence of TTX (1 µM) and picrotoxin (100 µM) to block inhibition and exclude any modulation coming from interneurons. We found a similar effect consisting in a shift of the cumulative probability distributions (Figure 4(e); *P* < 0.0001, K-S test. The frequency of mEPSCs was decreased by 53% when HCAR1 was activated compared to baseline condition (Figure 4(f); *P* = 0.009, one sample t test), while the amplitude was not altered (Figure 4(g); *P* = 0.63, one sample t test). The specificity of these effects was confirmed by the lack of agonist effects on mEPSCs recorded in neurons from HCAR1 KO mice (Figure 4(f), *P* = 0.64, and Figure 4(g), *P* = 0.47, one sample t test). These results provide evidence for a pre-synaptic action of HCAR1 on glutamatergic neurotransmission provided by MCs.

Some of our staining approaches and the published *in situ* hybridization (the Allen Brain Atlas, http://www.brain-map.org) are consistent with expression of HCAR1 by GCs. We therefore tested whether HCAR1 influenced GC excitability. Increasing current injection of 30 pA steps were applied until establishment of neuronal accommodation in baseline and during activation of HCAR1. The resulting maximum frequency of AP discharge achieved is reported in Figure 4(h). Result showed that the maximum firing frequency was decreased by about 20% under HCAR1 activation (*P =* 0.019, paired t-test). However, HCAR1 activation did not alter neuronal accommodation since the current injected to reach the maximum firing frequency was similar in both conditions (Figure 4(l), *P* = 0.14, paired t-test). GCs from HCAR1 KO mice were not affected by 3Cl-HBA (Figure 4(j), *P* = 0.62, paired t-test; Figure 4(k); *P* = 0.82, paired t-test). Finally, intracellular passive properties values of GCs showed no significant differences between baseline and HCAR1 activation (Supplementary Table 2).

These results suggest a marked pre-synaptic effect on the spontaneous release of glutamate at the MC-GC synapse and a mild reduction of GC excitability suggesting an expression of HCAR1 in GCs as well.

### HCAR1 activation in rat brain tissue modulates MC-GC synapses

**Figure 5.**
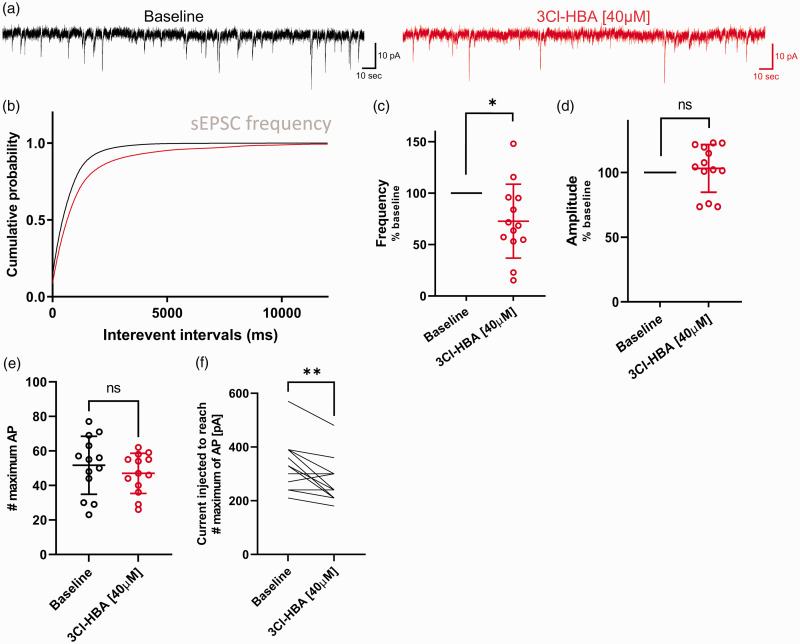
Granule cells from Sprague-Dawley rat decrease the sEPSC and firing frequency after activation of HCAR1. (a) Example traces of sEPSCs measure during baseline condition (black) and following application of 40 µM 3Cl-HBA (red). (b) Cumulative distributions of sEPSCs frequency were analyzed by using Kolmogorov-Smirnov test: *P* < 0.0001 versus HCAR1 activation. (c) Summary of recorded cells showing a decreased frequency of sEPSC by 27% induced by 3Cl-HBA compared to baseline (one sample t, n = 13, mean = 72.77 ± 35.96, *P* = 0.018). (d) sEPSC amplitude showed no statistically significant changes between baseline and 3Cl-HBA application (one sample t, n = 13, mean = 103.2 ± 18.38, *P* = 0.0049). Frequency and amplitude are shown in percentage compared to baseline. (e) Summary graph of the effect of HCAR1 activation on neuronal firing frequency following steps of current injection. The maximum number of action potentials evoked was calculated for each condition. No difference was observed (paired t test, n = 7, Baseline: mean = 51.69 ± 16.79, 3Cl-HBA: mean = 47 ± 11.59, *P* = 0.13). (f) The current injected to reach the maximum firing frequency was calculated for each condition. HCAR1 activation significantly decreases the current needed for reaching the maximum firing frequency (paired t test, n = 13, Baseline: mean = 327.7 ± 96.79, 3Cl-HBA: mean = 270 ± 80.31, *P* = 0.0049). Values are means ± SD, *P < 0.05, **P < 0.01, ***P < 0.001 versus 3Cl-HBA application.

The genetic distance between mice and rats is substantial, which goes along with significant differences at functional and behavioral levels.^33^ As rats are widely used in biomedical research and in order to obtain comparative information on the functional involvement of HCAR1, we next decided to move to the rat brain. We investigated the DG network in rat brain acute slices using the same approach as with mice, and recorded sEPSC from GCs (Figure 5(a)). We found that HCAR1 activation by 3Cl-HBA significantly altered the frequency of events, as indicated by the cumulative probability distributions (Figure 5(b); *P* < 0.0001, K-S test). The mean frequency of sEPSCs during HCAR1 activation was decreased by 27% compared to baseline (Figure 5(c); *P* = 0.018, one sample t test), while no change in amplitude was observed (Figure 5(d); *P* = 0.54, one sample t test).

We found that excitability of rat GCs was differently affected compared to mouse cells. Whereas the maximum firing frequency was not significantly different (Figure 5(e); *P* = 0.13, paired t-test), neuronal accommodation was reached at lower injected current steps during HCAR1 activation (Figure 5(f); *P* = 0.0049, paired t-test). Finally, basic electrophysiological properties of GCs did not show significant differences between baseline or HCAR1 activation (Supplementary Table 2).

These experiments in rat DG showed a pre-synaptic modulation of MC-GC synapses comparable with the mouse results. Overall, our results supports the functional presence of HCAR1 in the DG network and suggest a pre-synaptic location of the receptor, in line with recent papers reporting a modulation of CA1 and CA3 pyramidal cells in the rat hippocampus.^29,34^

### HCAR1 activation in epileptic human brain neurons

By down-modulating neuronal activity in rodent brains, HCAR1 represents an interesting target for tackling conditions of exuberant activity in humans such as found in the epileptic brain. The availability of human cortical tissue resected during surgery for relief of pharmacoresistant epilepsy gives a unique opportunity to investigate the physiology of cortical neurons and drug effects with obvious clinical relevance.

**Figure 6.**
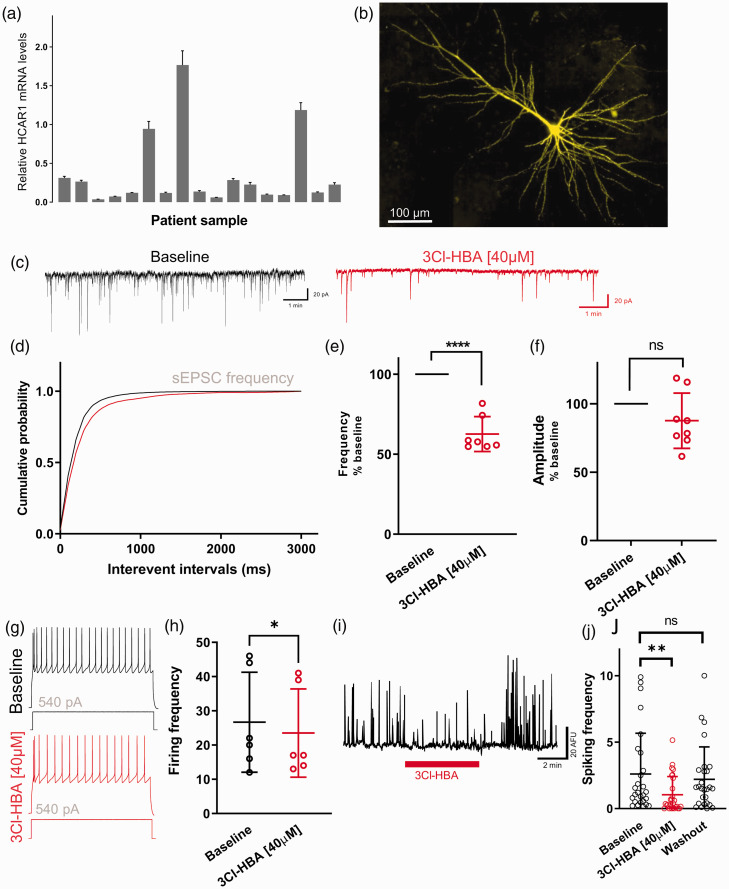
Cortical neurons from epileptic human patients decrease their spontaneous EPSC frequency following HCAR1 activation. (a) HCAR1 mRNA detection in human brain sample from 17 different pharmacoresistant epileptic patients undergoing resective surgery. Brain samples were from both male (57%) and female (43%) patients with median age 18 years, and originated from frontal (n = 5), insular (n = 2), parietal (n = 1), and temporal (n = 9) cortex from both hemispheres. Basal expression of HCAR1 is normalized to a reference gene (*β*-actin). All samples were run in triplicate and data is shown as means±SD. (b) Maximum intensity projection of a biocytin-filled recorded human cortical neuron. (c) Example traces of sEPSCs recorded during baseline condition (black) and following application of 40 µM 3Cl-HBA (red). (d) Cumulative distributions of sEPSCs frequency were analyzed by using Kolmogorov-Smirnov test: *P* = 0.0005 versus HCAR1 activation. (e) Summary of recorded cells showing a decreased frequency of sEPSC by ∼40% induced by 3Cl-HBA compared to baseline (one sample t, n_cells/patients_ = 7/5, mean = 62.54 ± 10.89, *P* < 0.0001). (f) sEPSC amplitude showed no statistically significant changes between baseline and 3Cl-HBA application (one sample t, n_cells/patients_ = 7/5, mean = 87.65 ± 20.2, *P* = 0.13). Frequency and amplitude are shown in percentage compared to baseline. (g) Representative traces from neurons recorded before and after HCAR1 activation obtained from a series of current injections (−120 to 540 pA, 2 sec, 30 pA increments); the response to 540 pA current injection is shown. (h) Summary graph of the effect of HCAR1 activation on neuronal firing frequency following steps of current injection (n = 6). The firing frequency was calculated from the number of AP evoked by 540 pA current injection during 2 seconds. Individual data and significance is shown between the baseline and 3Cl-HBA conditions (paired t test, n_cells/patients_= 6/5, Baseline: mean = 26.67 ± 14.62, 3Cl-HBA: mean = 23.5 ± 12.9, *P* = 0.035). (i) Example trace of calcium spiking activity from neurons in human acute slices with application of 3Cl-HBA. (j) Summary graph of spontaneous calcium spiking activity down-modulated by HCAR1 activation in cortical neurons from epileptic human patients (one-way ANOVA, n_cells/patients_= 27/5, Baseline: mean = 2.6 ± 3.07, 3Cl-HBA: mean = 1.04 ± 1.37, Washout: mean = 2.21 ± 2.44, *P* = 0.013). The calcium spiking activity of individual cells is shown. Values are means ± SD; *P* < 0.05, **P < 0.01, ***P < 0.001 versus 3Cl-HBA application.

First, we probed HCAR1 expression in the human cortex using qRT-PCR measurements. Figure 6(a) shows that HCAR1 mRNA transcripts were found in all 17 brain samples from patients that we tested. Three of them showed levels that were on average about nine fold higher than the average. We did not find a correlation with either gender or age in the samples, or with any other patient’s parameters available. Interestingly, these three samples originated from the temporal region.

We then performed both whole-cell patch clamp recordings and spontaneous neuronal calcium activity monitoring. Pyramidal cortical neurons were identified based on their morphology and electrical properties. The use of biocytin-containing pipette solutions followed by post-fixation and fluorescent streptavidin counter-staining of slices allowed verifying the morphology of these neurons (Figure 6(b)).

To examine neuronal modulation by HCAR1, we recorded sEPSC in control condition and under HCAR1 activation using 3Cl-HBA (Figure 6(c)). Results indicate a rightward shift of the interevent interval cumulative probability (Figure 6(d), *P* = 0.0005, K-S test), consistent with a decrease of the frequency of synaptic events by ∼40% (Figure 6(e), *P* < 0.0001, one sample t). No significant change in amplitude (Figure 6(f), *P* = 0.13, one sample t) or kinetics of sEPSCs was observed. Next, we looked at changes in excitability after activation of HCAR1 by injecting current steps of increasing amplitude. We quantified the number of AP discharge for 540 pA steps (Figure 6(g)), which revealed that excitability of these cells was decreased by about 12% compared to baseline after 3Cl-HBA application (Figure 6(h), *P* = 0.035, paired t test). No change in intracellular passive properties values of human pyramidal neurons between control and 3Cl-HBA bath application was detected (Supplementary Table 2).

We also recorded neuronal spiking activity in human slices using calcium imaging. Cortical neurons displayed spontaneous spiking activity, in some cases with periodic bursting of activity presumably reflecting the epileptic nature of the tissue. We tested for the sensitivity of these human neurons to the application of HCAR1 agonist. We observed that HCAR1 agonist 3Cl-HBA caused a significant and reversible reduction in calcium spiking activity in these neurons (Figure 6(l) and (j); P = 0.013, one-way ANOVA).

Taken together, these data suggest that neuronal activity is modulated by the activation of HCAR1 in acute brain slices obtained from epileptic patients. The receptor appears to act at the pre-synaptic level of the cortical network and affect excitability in recorded cells.

## Discussion

Evidence has accumulated over the past two decades indicating that lactate is more than a waste-product of metabolism. On the contrary, it is an important molecule used as metabolic fuel for a variety of cells and, more recently, as a signaling molecule,^3,8,16,35^ particularly since the discovery of a selective plasma membrane receptor coupled to G-proteins named HCAR1. In the present study, we demonstrate that HCAR1 is expressed in rodent and human brains and that its activation leads to a decrease in neuronal activity.

The precise functional role of HCAR1 in neurons has remained elusive because of the lack of specific antibodies that have hampered the reliable identification of HCAR1-positive cells. In this study, we instead used a mRFP reporter mouse. HCAR1 expressing cells was mainly found in the cerebellum and the hippocampus, where the positive cells predominantly correspond to hilar MCs. We did not find evidence for HCAR1 expression in interneurons or astrocytes. These results were confirmed using *in situ* hybridization technology (RNAscope™). MCs, via their communication with GCs, have been implicated in various forms of mnemonic functions such as associative memory,^36^ pattern separation,^37^ and recall of memory sequences.^38^ Systemic administration of D-lactate and of the HCAR1 agonist 3,5-DHBA 15 min before performing inhibitory avoidance training showed memory impairments compared to saline administration, suggesting the involvement of HCAR1 signaling in learning and memory.^9^ However, whether HCAR1 is involved in the dentate gyrus mnemonic processes is not yet known.

Previous studies by us^24,27^ and others^29,34^ reported evidence for a neuromodulatory action of HCAR1 on neurons. Based on our histological observations and our functional characterization in cultured neurons, we asked whether HCAR1 would act as neuromodulator of DG neurons *in situ*. Hilar MCs provide broadly distributed excitatory outputs to a large number of GCs.^32^ Using electrophysiology recordings in the mouse hippocampus, we found that dentate GCs displayed a reduction in mEPSC and sEPSC frequency without changes in synaptic event amplitude. This result suggests presynaptic control of spontaneous neurotransmitter release at MC-GC, consistent with our description of HCAR1-expressing cells. These results could be replicated in rats, and indicate that HCAR1 is also present and functional in the DG, where GC synaptic events were markedly reduced by HCAR1 activation.

This result brings new perspectives for the understanding of neuronal activity at MC-GC synapses. These circuits are critically involved in temporal lobe epilepsy,^32,39,40^ where MCs either become overactive and driving GC firing,^41^ or undergo cell death, reducing the magnitude of feed-forward inhibition.^42^ Therefore, by acting on HCAR1, lactate may be beneficial to temporal lobe epilepsy management, an idea also supported by experiments showing the capacity of 3.5-DHBA to successfully decrease spiking frequency in hippocampal subiculum neurons in an *in vitro* model of epilepsy using 4-aminopyridine.^43^ It was also demonstrated that MCs are more susceptible to excitotoxicity than GCs,^44^ therefore lactate could exert a dual role of metabolic neuroprotective support and of signaling molecule acting on HCAR1 to lower activity. Whether MCs metabolize lactate has not been directly addressed to our knowledge. However, the lactate transporter MCT2 appears to be present in hilar neurons,^45,46^ suggesting that they are equipped to use lactate as a metabolic substrate. Even though most aCSF solutions used for *in vitro* slice electrophysiology contain high concentrations of glucose (10–25 mM), we chose to use 2.5 mM glucose, as found in the brain *in vivo,*^47^ reasoning that this low glucose level should prevent excessive lactate production by astrocytes that could interfere with HCAR1 signaling. Knowing the vulnerability of MCs,^44^ pyruvate (3–5 mM) was supplemented throughout the experiment to support neuronal metabolic needs and to bring protective antioxidant effects, as shown before.^48^

We did not find unequivocal HCAR1 expression in GCs, which will needs further investigations to be established. However, our experiments revealed the functional involvement of HCAR1 in diminishing mouse GC excitability. Rat GCs followed a similar trend. In support of this idea, it has been shown that paired-pulse ratio and coefficient of variation (CV^−2^) of CA3 pyramidal cell in rats evoked on mossy fiber stimulation are transiently decreased by lactate perfusion.^34^ Change in the CV^−2^ is indicative of a presynaptic modulation. Because mossy fibers consists of axons projecting from GCs to CA3,^49^ this result would be compatible with the functional presence of HCAR1 in GCs.

Overall, HCAR1 was shown to be functionally present in rodent hippocampus. In rat CA1 pyramidal neurons excitability is modulated by lactate and HCAR1 agonist.^29^ Rat subicular neuronal activity is reduced by HCAR1 activation.^43^ CA3 pyramidal neurons are also modulated by lactate or HCAR1 agonist; however, they appear to express another putative lactate-sensitive receptor coupled to a different effector system.^34^ In the present study, we show that in the DG, HCAR1 is mainly present in hilar MCs and acts on GCs.

To our knowledge, we present here the first report of functional effects of HCAR1 activation in fresh human brain tissue. Indeed, while known to be present in human peripheral tissue, HCAR1 expression and activity in human brains was unknown. Rodent models, particularly mice, are widely used in biomedical research notably due to the ability to manipulate their genome and to the fact that many mechanisms and paradigms translate well between species. Nevertheless, in translational research, over 90% of neurological drug candidates with promising animal results have failed in human clinical trials,^50^ highlighting the crucial importance to validate rodent results with human tissue. Thanks to our access to fresh brain tissue from patients undergoing surgical resections of epileptic foci, we could determine that all human tissue samples contained HCAR1 mRNA transcripts. However, we faced obvious limitations in working with human samples, which include that one cannot compare HCAR1 expression levels in healthy non-epileptic brains, and that the patient individual epileptic condition, the medication received, and the neurosurgical procedure may also influence HCAR1 expression. By preparing acute brain slices from brain tissue blocks of patients, we found both by whole-cell patch-clamp and by calcium imaging that activating HCAR1 caused a down-modulation of spontaneous activity. However, we observed that among all cells analyzed after calcium imaging, 20% of them showed a moderate increased activity under application of the HCAR1 agonist 3Cl-HBA. This effect could be explained by the putative existence of other lactate receptors with G_s_ activity instead of the HCAR1 G_i_ activity as proposed by others,^16,34^ or could involve the additional G_βγ_ action of HCAR1 observed in primary neurons.^27^

In conclusion, we show consistent effects of HCAR1 activation in the rodent DG as well as human neurons from pharmacoresistant epileptic patients. In addition to being a promising new target for the development of anti-epileptic drug treatment, we can propose that HCAR1 could serve the role of providing a metabolic readout for neurons to tune their activity according to the metabolic state during brain or physical activity.

## Supplemental Material

sj-pdf-1-jcb-10.1177_0271678X221080324 - Supplemental material for Activation of lactate receptor HCAR1 down-modulates neuronal activity in rodent and human brain tissue Click here for additional data file. Supplemental material, sj-pdf-1-jcb-10.1177_0271678X221080324 for Activation of lactate receptor HCAR1 down-modulates neuronal activity in rodent and human brain tissue by Marc Briquet, Anne-Bérengère Rocher, Maxime Alessandri, Nadia Rosenberg, Haissa de Castro Abrantes, Joel Wellbourne-Wood, Céline Schmuziger, Vanessa Ginet, Julien Puyal, Etienne Pralong, Roy Thomas Daniel, Stefan Offermanns and Jean-Yves Chatton in Journal of Cerebral Blood Flow & Metabolism
