# Supplementary material for: Activation of lactate receptor HCAR1 down-modulates neuronal activity in rodent and human brain tissue
Source: J Cereb Blood Flow Metab. 2022 Mar 3;42(9):1650–65. doi: 10.1177/0271678X221080324 (PMC9441721; doi:10.1177/0271678X221080324)
Supplement: sj-pdf-1-jcb-10.1177_0271678X221080324 - Supplemental material for Activation of lactate receptor HCAR1 down-modulates neuronal activity in rodent and human brain tissue [file sj-pdf-1-jcb-10.1177_0271678X221080324.pdf]

Supplementary Information for

Activation of lactate receptor HCAR1 down-modulates neuronal activity in rodent and human brain tissue

Marc Briquet [1], Anne-Bérengère Rocher [1], Maxime Alessandri [1], Nadia Rosenberg [1], Haissa de Castro Abrantes [1], Joel Wellbourne-Wood [1], Céline Schmuziger [1], Vanessa Ginet [1], Julien Puyal [1], Etienne Pralong [2], Roy Thomas Daniel [2], Stefan Offermanns [3], Jean-Yves Chatton[1,4,*]

[1]Department of Fundamental Neurosciences, University of Lausanne, Switzerland; [2]Department of Neurosurgery Service, University Hospital of Lausanne and faculty of Biology and medicine, UNIL, Lausanne, Switzerland; [3]Max Planck Institute for Heart and Lung Research, Bad Nauheim, Germany; [4]Cellular Imaging Facility, University of Lausanne, 1005 Lausanne, Switzerland

Supplementary Material and Methods

Organotypic culture. Organotypic hippocampal slice cultures were prepared from 3 day-old WT mice. After decapitation, brains were quickly removed and plunged into ice-cold filtered dissection medium containing 50% MEM, 1% Penicillin-Streptomycin (Invitrogen, catalog # 15140122) and 10 mM Tris (Merck-Millipore, catalog # 8382). The hippocampi were rapidly and carefully dissected out and put both on Teflon-disk in order to obtain 400 µm-thick transversal section using tissue-cutter (McIlwain Tissue Chopper, TC752). Slices were placed onto porous hydrophilic LCR membrane (Merck-Millipore, catalog # FHLC04700) in the Millicell-insert (Merck-Millipore, catalog # PICM03050), which were transferred into 35mm Petri dish. Each Petri dish contained 1mL of pre-warmed culture medium composed of filtered 50 % MEM, 25% horse serum (Invitrogen, catalog # 26050-047), 25% HBSS, 7.5% $NaHCO_3$, 1% Penicillin-Streptomycin, 36mM D-Glucose and 5mM Tris, Petri dish containing the slices were incubated during 4 days at 36°C and then transferred into a 33°C incubator for the following weeks. Culture medium was first changed after 24h and then every 3-4 days. Widefield calcium imaging was performed with an upright epifluorescence microscope (FN1, Nikon, Tokyo, 163 Japan) using a 40X 0.8 N.A water-immersion objective lens. Fluorescence excitation wavelengths were selected using a fast filter wheel (Sutter Instr., Novato, CA) and fluorescence was detected using an Evolve EMCDD camera (Photometrics, Tucson, AZ, USA). Digital image acquisition and time series were computer-controlled using the Metafluor software (RRID:SCR_014294)

Supplementary Table 1. Summary of primers used for qRT-PCR in human and mouse brain experiments.

**Human**	HCAR1	GCCCAGCACTGTTTACCTTTTC	CCCCAAAAGCCCAGTGTCTAC
	β-actin	CTGTACGCCAACACAGTGCT	GCTCAGGAGGAGCAATGATC
**Mouse**	HCAR1	GGGGACTGTGTATCTTCTGA	GAGTCTTGGTGTAGAATTTGG
	GAPDH	TCCATGACAACTTTGGCATTG	CAGTCTTCTGGGTGGCAGTGA

Supplementary Table 2. HCAR1 activation did not affect passive properties of cortical human neurons and rodent GCs.

	Human		Mouse		Rat	
	Baseline	3Cl-HBA	Baseline	3Cl-HBA	Baseline	3Cl-HBA
Rheobase	351 ± 83.6	357.2 ± 71.4	52.2 ± 28.9	55.1 ± 31.6	92.9 ± 33.2	89.4 ± 41.4
RMP	-73.8 ± 3.2	-74.4 ± 2.2	-74.2 ± 11.5	-70.6 ± 10.6	-74.3 ± 4.7	-74.5 ± 5
R_N	68.2 ± 17.1	72.1 ± 12.2	235.9 ± 65.7	235.9 ± 56.6	207.8 ± 52	222.6 ± 59.8

All parameters were tested in all cells used for analysis. 3Cl-HBA was applied at 40µM. Data are shown as means±SD and analysis was done using one sample t-test. RMP, resting membrane potential; R_N, input resistance.

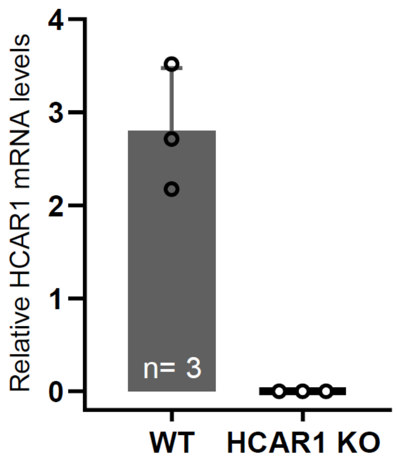

Supplementary Figure 1. HCAR1 mRNA detection in hemibrain after dissection of 1-month old mice. Results showed no expression of HCAR1 mRNA transcript in HCAR1 KO samples (WT: 2.8±0.68; KO: 0). Results are expressed relative to GAPDH expression as means±SD. The number of experiments are indicated in the graph.

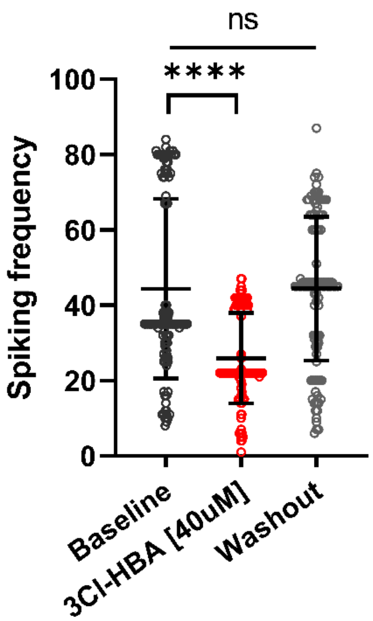

Supplementary Figure 2. Hippocampal neurons from mouse organotypic slices decrease their spiking frequency after activation of HCAR1. Summary graph of spontaneous calcium spiking activity down-modulated by HCAR1 activation by 3Cl-HBA (40µM) in hippocampal neurons from WT mice (one-way ANOVA, n= 107 cells from 5 experiments, Baseline: mean = 44.41 ±,23.87, 3Cl-HBA: mean = 25.99 ± 12, Washout: mean = 44.44 ± 19.13, P < 0.0001). The calcium spiking activity of individual cells is shown. Values are means ± SD; P < 0.05, **P < 0.01, *** P < 0.001, **** P < 0.0001 versus 3Cl-HBA application and washout.
